# Disseminated Leishmaniasis Due to Using Immunosuppression Drugs: A Case Report

**Published:** 2020

**Authors:** Maryam AFSHOON, Mohammadreza ABDOLSALEHI, Golnaz ALINIA, Katayoun BORHANI, Bahareh YAGHMAIE, Mahmoud KHODABANDEH

**Affiliations:** 1. Pediatric’s Center of Excellence, Children’s Medical Center, Tehran University of Medical Sciences, Tehran, Iran; 2. Department of Infectious Diseases, Pediatric’s Center of Excellence, Children’s Medical Center, Tehran University of Medical Sciences, Tehran, Iran

**Keywords:** Visceral leishmaniasis, Splenomegaly, Pancytopenia, Iran

## Abstract

Visceral leishmaniasis is a common parasitic disease between humans and animals, transmitted by sandflies (*Phlebotomus*) in the Mediterranean countries, including Iran. The statistics have been reported less than real due to errors in the diagnosis and reporting of affected cases. In this report, we will present the symptoms and manifestations of this disease to reduce late detection and exacerbating factors. The patient was a three-year-old girl from Tehran, Iran who had ascites and hepatomegaly. When she was 9 month-old, she was diagnosed as autoimmune hepatitis after liver biopsy and she was treated with immunosuppressive drugs (Azathioprine, prednisolone, and cyclosporine) for 22 months, but later she suffered from fever, pancytopenia, and hepatosplenomegaly. Then a bone marrow biopsy was done for her. There was a large amount of Leishman body in her bone marrow and treatment for Kala-azar was started for her. In patients with prolonged fever and splenomegaly, especially association with pancytopenia, consider leishmaniasis. Immunosuppressive drugs can disseminate parasitic diseases, including visceral leishmaniasis.

## Introduction

Leishmaniasis is a common disease between humans and animals transmitted by the protozoa (*Leishmania*). The intracellular type of Leishmania is called amastigote and its extracellular type is called promastigote. It is transmitted by sandflies (*Phlebotomus*) and it limited to the skin, mucus, or visceral lesions (1). Clinical symptoms of visceral leishmaniasis are often confusing because their symptoms are different and it associated with hematologic disorders (2).

The incubation period varies from weeks to months, and symptoms include prolonged and low-grade fever, irregular pattern of fever, weight loss, hepatomegaly and splenomegaly, and pancytopenia. The disease begins with fever and agitation and it continues with weight loss and splenomegaly and hepatomegaly, and finally will lead to death in 2–3 years. The cause of death is usually secondary infections and internal bleeding (3). Decreased platelets in patients sometimes cause gastrointestinal bleeding (4). Liver biopsy, spleen biopsy, or bone marrow aspiration are the golden diagnostic methods of kala-azar (5). Visceral leishmaniasis has been reported occasionally in all of 31 provinces of Iran.

However, this disease is endemic in some parts of the country such as northwestern and southern areas with an annual rate of 300 new cases (6). If the disease is not diagnosed for treatment, it may lead to deaths up to 98% of cases, especially in children (1).

## Case Report

The patient was admitted to Children’s Medical Center of Tehran in 2019 due to ill health with an undiagnosed condition. All the biopsy samples and other clinical investigations performed were part of routine clinical investigative procedures to determine the nature of the illness.

This report does not contain any identifiable information that could be used to compromise patient confidentiality. Informed consent was taken from the parents before the study.

The patient was a three-yr-old girl who at 9 months of age, one week after traveling to Meshkin-Shahr district in north of Ardabil Province, Northwest of Iran, had a high fever in nights (39–40 °C) and with restlessness. She was referred to the local hospital and conservative treatment was done for her, then she was discharged. Four weeks later, she returned to the hospital with fever. On examination, several macular lesions were seen in the forearm, and in lab tests, hepatic enzymes were raised. In examination and ultrasonography, she had ascites and hepatosplenomegaly. The ascites fluid was tapped and analyzed, which was normal. Due to continuing fever, the patient became a liver biopsy. Liver biopsy was reported autoimmune hepatitis grade 1 and stage 1. She was treated with Azathioprine and prednisolone tablets. Three months later, hepatic enzymes were normal and corticosteroid was tapered. Due to increased liver enzymes, cyclosporine was begun for her.

Several months later, the patient referred with Fever Unknown Origin (FUO), additional lab testing was performed for her. All of the lab tests were normal but three weeks later, in her lab test, pancytopenia occurred (Table 1).

**Table 1:** Laboratory findings of the patient

***Laboratory Tests***														
Blood test														
WBC	HB	PLT	ESR	CRP	ALT	AST	ALK-P	ALB	Protein	BS	BUN	Cr	Na	PBS
1990	7.8	54000	85	63	75	26	499	1.7	6.5	77	6	0.4	131	Normal
Urine Analysis														
SG		PH		Pro	Nitrite		RBC	WBC		Leukocyte esterase				
1013		6		Positive	Positive		8 –10	10–15		Positive				

**Fig. 1:**
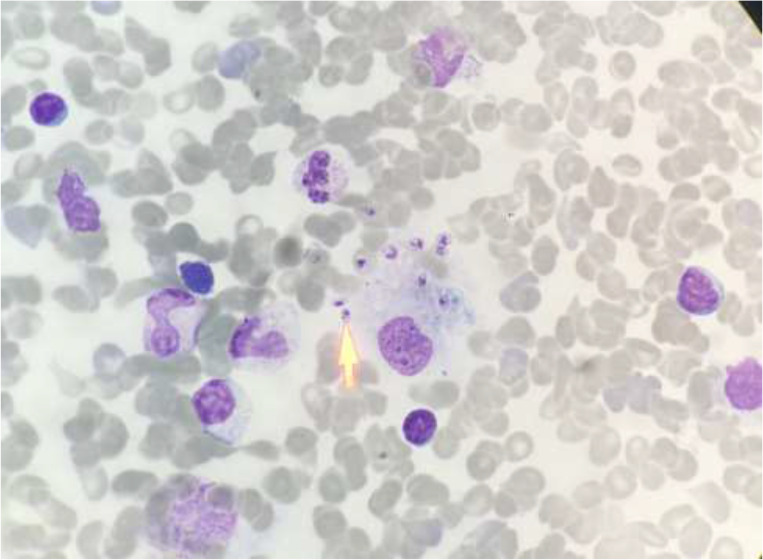
Giemsa-stained bone marrow smear prepared from patient’s bone marrow. The arrow indicates one of Lishman bodies (Original picture)

Marrow biopsy was done for her, and many Leishman bodies were seen in her marrow biopsy (Fig. 1). The patient was treated with amphotericin liposomal in days 1, 2, 3, 4, 5, 14, and 21. After that, the fever was discontinued and pancytopenia improved and spleen size decreased.

## Discussion

The current study reported a case presented with high fevers at nights, restlessness, ascites and hepatomegaly which was later diagnosed as Kala-azar leishmaniasis. In a study in Mexico, a 5-year-old girl with a 4-month history of fatigue and a 39 °C intermittent fever and a 7 kg weight loss with enlargement of the spleen, kala-azar was diagnosed (7). Therefore, the symptoms are important for diagnosis.

In a study in Iran, on 217 hospitalized patients with DAT positive, sign and symptom were paleness, splenomegaly, fever, hepatomegaly, and lymphadenopathy (8). In our study, the patient had fever and hepatosplenomegaly and pancytopenia.

In France, a two-year-old male with fever and splenomegaly was evaluated, the result of his bone marrow biopsy, spleen, and specific serology was negative, but after three months, his biopsy of bone marrow and specific Leishmaniasis serology was positive (9). In France, the relation between ALL and visceral leishmaniasis was studied. Visceral leishmaniasis can be considered as a rare and opportunistic infection in ALL (10). Immunosuppressive drugs and Immunodeficiency can disseminate leishmaniasis.

Another case report in Italy was presented to the child with symptoms and abnormal blood test results, suspected to ALL. His final diagnosis was leishmaniasis and successfully was treated (11). This report considers visceral leishmaniasis to be a differential diagnosis of ALL children, especially in children who live or travel in the endemic areas.

In India, common symptoms of leishmaniasis include fever, hepatosplenomegaly, anemia, leukopenia, thrombocytopenia, pancytopenia, histiocytosis, and DIC, especially in endemic areas (12). Pancytopenia and hepatosplenomegaly were prominent in the patient in our study and she had a history of traveling to endemic areas.

In Iran, from 516 kala-azar cases: 50.6% of patients were from Meshkin-Shahr and Moghan (13). Our patient had a travel history to Meshkin-Shahr.

Visceral leishmaniasis can be neglected due to the commonality of symptoms with some hematologic diseases such as ALL, as well as some diseases, including immunodeficiency and the use of immunosuppressive drugs such as corticosteroid exacerbation of leishmaniasis. Especially if there is a history of travel to endemic areas.

If there are common symptoms with underlying illness, leishmaniasis may be detected late, and even death, especially in children. This parasite sometimes cannot be detected in the early stages of the disease in tissue sampling, but if there is a strong clinical suspicion, it is recommended to repeat the tests. Therefore, it is important to get a history of the long fever, splenomegaly, pancytopenia, and travel to endemic areas.

## Conclusion

In patients with prolonged fever and splenomegaly, especially association with pancytopenia, must be considered leishmaniasis as an important differential diagnosis. Therefore, it is important to ask about the history of travel to endemic areas, history of medication and underlying disease
